# Near-Infrared Spectral Similarity between Ex Vivo Porcine and In Vivo Human Tissue

**DOI:** 10.3390/life13020357

**Published:** 2023-01-28

**Authors:** Eva de Vries, Lejla Alic, Rutger M. Schols, Kaj S. Emanuel, Fokko P. Wieringa, Nicole D. Bouvy, Gabriëlle J. M. Tuijthof

**Affiliations:** 1 Research Engineering, Faculty of Health, Medicine, Life Sciences, Maastricht University, 6229 ER Maastricht, The Netherlands; 2 Magnetic Detection and Imaging Group, Technical Medical Centre, Faculty of Science and Technology, University of Twente, 7522 NB Enschede, The Netherlands; 3 Department of Plastic, Reconstructive and Hand Surgery, Maastricht University Medical Centre+, 6229 HX Maastricht, The Netherlands; 4 Department of Orthopaedic Surgery, Faculty of Health, Medicine, Life Sciences, Maastricht University, 6229 ER Maastricht, The Netherlands; 5 Department of Orthopedic Surgery and Sports Medicine, Amsterdam UMC, University of Amsterdam, Amsterdam Movement Sciences, 1105 AZ Amsterdam, The Netherlands; 6 IMEC, Holst Centre, 5656 AE Eindhoven, The Netherlands; 7 Department of Surgery, School for Nutrition, Toxicology and Metabolism, Maastricht University Medical Centre+, 6229 HX Maastricht, The Netherlands; 8 Department of Biomechanical Engineering, Faculty of Engineering Technologies, University of Twente, 7522 NB Enschede, The Netherlands

**Keywords:** diffuse reflectance spectroscopy, adipose tissue, nerve tissue, classification, spectra

## Abstract

Background: In vivo diffuse reflectance spectroscopy provides additional contrast in discriminating nerves embedded in adipose tissue during surgery. However, large datasets are required to achieve clinically acceptable classification levels. This study assesses the spectral similarity between ex vivo porcine and in vivo human spectral data of nerve and adipose tissue, as porcine tissue could contribute to generate large datasets. Methods: Porcine diffuse reflectance spectra were measured at 124 nerve and 151 adipose locations. A previously recorded dataset of 32 in vivo human nerve and 23 adipose tissue locations was used for comparison. In total, 36 features were extracted from the raw porcine to generate binary logistic regression models for all combinations of two, three, four and five features. Feature selection was performed by assessing similar means between normalized features of nerve and of adipose tissue (Kruskal–Wallis test, *p* < 0.05) and for models performing best on the porcine cross validation set. The human test set was used to assess classification performance. Results: The binary logistic regression models with selected features showed an accuracy of 60% on the test set. Conclusions: Spectral similarity between ex vivo porcine and in vivo human adipose and nerve tissue was present, but further research is required.

## 1. Introduction

Intraoperative nerve localization embedded in adipose tissue is important in a large variety of surgical procedures to prevent iatrogenic nerve injury. This can be particularly challenging using human eyesight and haptic feedback, due to the presence of inter-individual anatomic variation and limited haptic feedback provided by the nerves [1,2,3]. Failure to identify the nerves can lead to iatrogenic injury. A potentially devastating complication, which can result in both sensory and motor function loss, the detection rate of the recurrent laryngeal nerve in only 158 of 192 patients led to six serious iatrogenic injuries [4], and a retrospective analysis of 722 surgical cases found 17.4% sustaining iatrogenic nerve injury [5]. Various intra-operative optical imaging techniques have been proposed to identify critical tissues [2,6,7]. Diffuse reflectance spectroscopy inherently has the advantage of being non-invasive and requires tissue contact only for a short period to allow for surface scanning of tissue [8]. Although hyperspectral imaging is not a routine surgical tool, we have shown its feasibility to distinguish between nerve, lymph, muscle, and adipose human tissues based upon tissue-specific optical reflectance signatures [1,9,10]. Our studies confirmed that indium gallium arsenide (InGaAs) sensors are better suited for discrimination between nerves and surrounding adipose tissue than Si sensors [1,10]. A wide band spectrometer (350–1830 nm) identifies reflective features for water and lipids present in these tissue types [11,12]. One general challenge in achieving clinically acceptable classification performance is the presence of sufficient data to generate predictive logistic regression models. Therefore, we aimed to assess the feasibility of substitute ex vivo porcine tissue as input data. We argue that ex vivo porcine tissue is abundantly available, easier to process, requires less strict ethical regulation, and does not hamper the surgical workflow. Porcine tissue was selected as a potential alternative for human tissues as its physiological, morphological, histological, and biomolecular characteristics demonstrate similarity to humans [13,14,15] (Table 1).

The goal of this study was to assess whether ex vivo porcine tissue could generate substitute input data to train models for classification of in vivo human tissue types. To this end, the spectral similarity of ex vivo porcine spectral data and in vivo human data of nerve and adipose tissue was assessed, and binary logistic regression models were generated with porcine spectra and validated with human data.

## 2. Materials and Methods

### 2.1. Subsection

**Figure 1:**
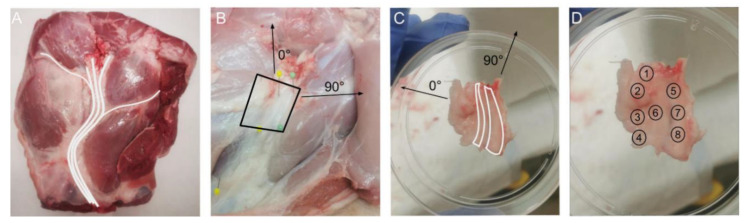
Pictorial assay for obtaining porcine samples. (**A**) Identification of the brachial plexus nerve in prescapular region (porcine shoulder) and sample planning. (**B**) Dissection of one sample. (**C**) Orient sample and digital mapping nerve locations. (**D**) Digital documentation of the 5 to 10 distinct locations measured on the sample.

A total of 44 samples were obtained post-mortem from the prescapular region of seven Sus scrofa domesticus female pigs (aged between 3 and 6 months old). The samples had an area of approximately 30 × 30 mm, with thickness of 1 mm, and consisted predominantly of adipose tissue containing one or more nerves of the brachial plexus (Figure 1). Within 30 min of retrieval, the samples were labelled and stored at −80°C until measurement. Prior to the measurements, the samples were digitally mapped using pictures of the tissue samples and the physical samples presented in the same orientation. As illustrated in Figure 1, the mapping produced the point locations of adipose and of nerve tissue (Figure 1). The tissues were mapped by a circle as follows: adipose tissue if the layer was sufficiently thick (visually inspected by lack of opacity) and nerve tissue if the diameter was >2 mm.

### 2.2. Experimental Setup and Measurements

A previously reported experimental setup [1,10] was used consisting of a custom made 2 mm optical fiber probe (TNO, Eindhoven the Netherlands and Light Guide Optics, Rheinbach, Germany), a modified Xenon light source (D-light C, Karl Storz, Tuttlingen, Germany), and a spectrometer (Analytical Spectral Devices, Inc., Boulder, CO, USA) covering the range 350–1830 nm with a spectral resolution of 1 nm [19]. All spectra were calibrated by a Spectralon spectrum with 50% light intensity correcting for the spectral non-uniformity of the light source and influence of the dark current [20]. Prior to data acquisition, the samples were thawed at room temperature using a sterile PBS-soaked gauze for 10 min. The spectral measurements were acquired with the Indico Software (version 5.5) on each mapped location by placing the probe tip perpendicular on the sample surface using gentle pressure. Between the measurement locations, the probe tip was wiped clean with an ethanol-soaked sterile gauze and allowed to dry. Acquired ex vivo porcine spectra were labelled according to the tissue type using digital pre-mapping (Figure 1). The in vivo spectra of human adipose and nerve tissue were acquired previously by the same protocol [1,10].

### 2.3. Data Processing

Both ex vivo porcine spectra and in vivo human spectra [1,10] were normalized according to the modified Bauer equation [21]:

(1) $$S_{n,\;corrected}\left(\lambda \right)=\frac{S_{n,\;raw}\left(\lambda \right)}{S_{w50\%,\;raw}\left(\lambda \right)}\cdot \frac{50\%}{100\%}$$

with *S* as the spectral data as functions of the wavelengths λ. All data processing was performed by in-house developed and existing algorithms (using MATLAB environment 2020, MathWorks, Inc., Natick, MA, USA) [1]. Similarity between the entire porcine and human diffuse reflectance spectral data was quantitatively assessed by the normalized maximum correlation coefficient (NMCC), and the normalized root mean squared error (NRMSE) [22]. The NMCC is a measure of shape similarity, and is calculated as:

(2) $$NMCC=\frac{max\left|\left(S_{Pn,\;corrected}*S_{Hn,\;corrected}\right)\left[\lambda \right]\right|}{\sqrt{\sum_{i=\lambda }^{m}S_{Pn,\;corrected}{\left(\lambda \right)}^{2\;}}\cdot \sqrt{\sum_{i=\lambda }^{m}S_{Hn,\;corrected}{\left(\lambda \right)}^{2}}}$$

With *P* representing porcine spectra, *H* representing human spectra, $(S_{Pn,\;corrected}*S_{Hn,\;corrected})\left[\lambda \right]$ is the cross correlation of the spectral curves of porcine and human tissue, and *m* is the total number of wavelengths (*m* = 1480). The NRMSE is the absolute difference between signals, and is calculated as:

(3) $$NRMSE=\frac{\sqrt{\sum_{i=\lambda }^{m}{\left(S_{Pn,\;corrected}\left(\lambda \right)-S_{Hn,\;corrected}\left(\lambda \right)\right)}^{2\;}}}{\max(S_{Pn,\;corrected}\left(\lambda \right))-\min(S_{Pn,\;corrected}\left(\lambda \right))}$$

Supervised classification as adipose or nerve tissue was performed by the binary logistic regression models (BLRM). To this end, 36 spectral features were extracted from the diffuse reflectance spectral data [1,9,10,19]. These features were previously reported as characteristic for absorption features of blood, water, and fat [23,24,25]. They were automatically extracted from the in vivo human data [1,9,10,19] and the ex vivo porcine data. To prevent possible model inaccuracies, feature scaling was performed where all features were individually normalized per underlying tissue type using the z-score normalization (which produces zero mean at a unit variance features [26]):

(4) $$Feat_{n,norm}=\frac{Feat_{n,org}-\mu_{n,org}}{\sigma_{n,org}}$$

where *Feat_n,org_* is the actual feature value corresponding to a specific tissue type (nerve or adipose) and a source (porcine or human), *µ_n,org_* is the mean of that feature over the entire porcine (or human) dataset, and *σ_n,org_* is the standard deviation for that feature over the entire porcine (or human) dataset. 

The first classification step involved feature selection based upon statistical similarity between normalized ex vivo and in vivo features. Since not all features showed a normal distribution, this was assessed by the Kruskal–Wallis test. The mean values were considered similar if *p* ≥ 0.05.

Second, to prevent overfitting, the binary logistic regression models (BLRMs) were trained using labelled (adipose or nerve) data by applying forward feature selection up to five features: for two features (276 combinations), for three features (2024 combinations), for four features (10626 combinations), and for five features (42,504 combinations). The BLRMs were trained using 80% of the ex vivo porcine data and were cross validated with the remaining 20%. The best performing BLRMs for each set of features (for 2, 3, 4, and 5 features) were selected by the maximum accuracy on the cross validation set. The accuracy of these BLRMS was tested by the in vivo human data. Accuracy was defined as the percentage of tissue samples correctly identified as nerve or adipose as predicted by the BRLMs. 

## 3. Results

**Figure 2:**
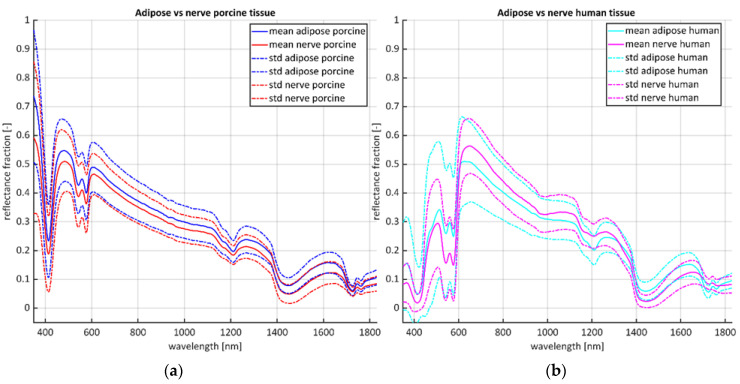
Mean calibrated spectra (solid lines) flanked by the standard deviation (striped lines). (**a**) Ex vivo adipose porcine (blue) vs. nerve porcine tissue (red). (**b**) In vivo adipose human (cyan) vs. human nerve tissue (magenta).

A total of 1375 porcine spectra originating from 44 ex vivo porcine samples (124 nerve, 151 adipose) were acquired and compared to previously published data [1,10] consisting of 55 in vivo human spectra (32 nerve, 23 adipose) originating from 24 patients undergoing thyroid (19 patients) or carpal tunnel release surgery (5 patients). The calibrated spectra per source show differences in tissue types, with the average spectra of adipose tissue showing generally a larger reflectance magnitude compared to the nerve tissue for the porcine source, with the reversed being present for the human source (Figure 2).

**Figure 3:**
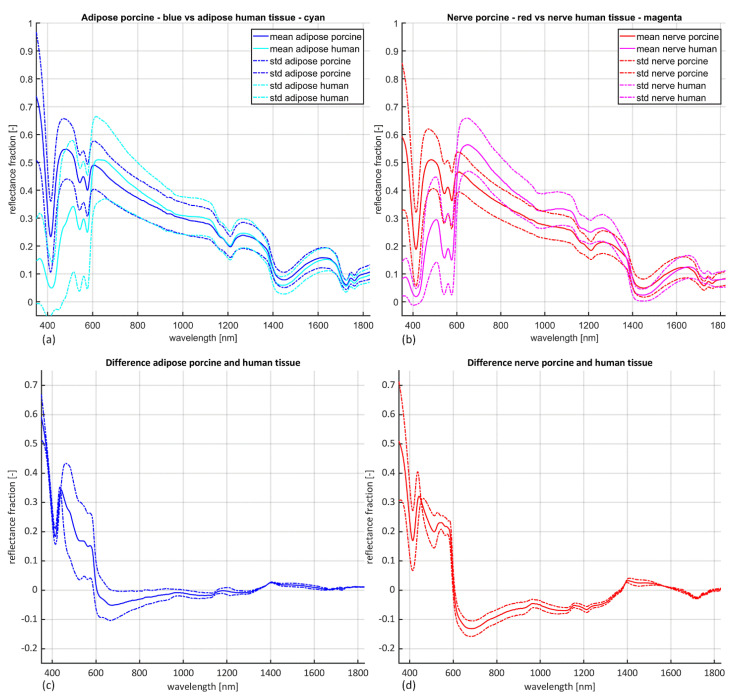
Mean calibrated spectra (solid lines) flanked by the standard deviation (striped lines). (**a**) Porcine (blue) vs. human adipose tissue (cyan). (**b**) Porcine (red) vs. human nerve tissue (magenta). (**c**) Difference between porcine and human adipose tissue. (**d**) Difference between porcine and human nerve tissue.

Visual inspection of the calibrated spectra per tissue type (Figure 3) shows similarities in the shape and amplitude of the spectral reflectance for adipose as well as nerve tissue when comparing the ex vivo porcine and in vivo human data. Relative to the mean human spectral reflectance magnitudes, the porcine reflectance is generally higher for wavelengths 350–600 nm and > 1350 nm (Figure 3 bottom), and lower for wavelengths between 600–1350 nm. This is consistent for both tissue types. NMCC values were found larger than 0.9 for 99% of adipose tissue (with 50% a value ≥ 0.95), and for 99.5% of nerve tissue (with 40% a value ≥ 0.95). NRMSE values were found smaller than or equal to 0.1 for 0.5% of adipose tissue (with 21% a value ≤ 0.2), and for 1.5% of nerve tissue (with 19% a value ≤ 0.2).

**Figure 4:**
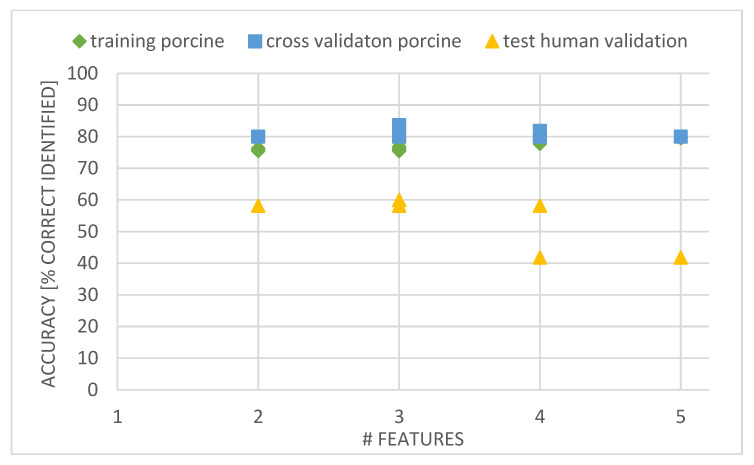
Performance of the BLRMs generated with combinations of 2, 3, 4 and 5 features, respectively for the 24 features showing similar means with human data and showing the highest performance on cross validation set. Green: accuracy of the training set. Blue: accuracy of the cross validation set. Yellow: accuracy of the human test set. Note that each dot, square and triangle can represent multiple BLRMs which give the same classification performance.

**Figure 5:**
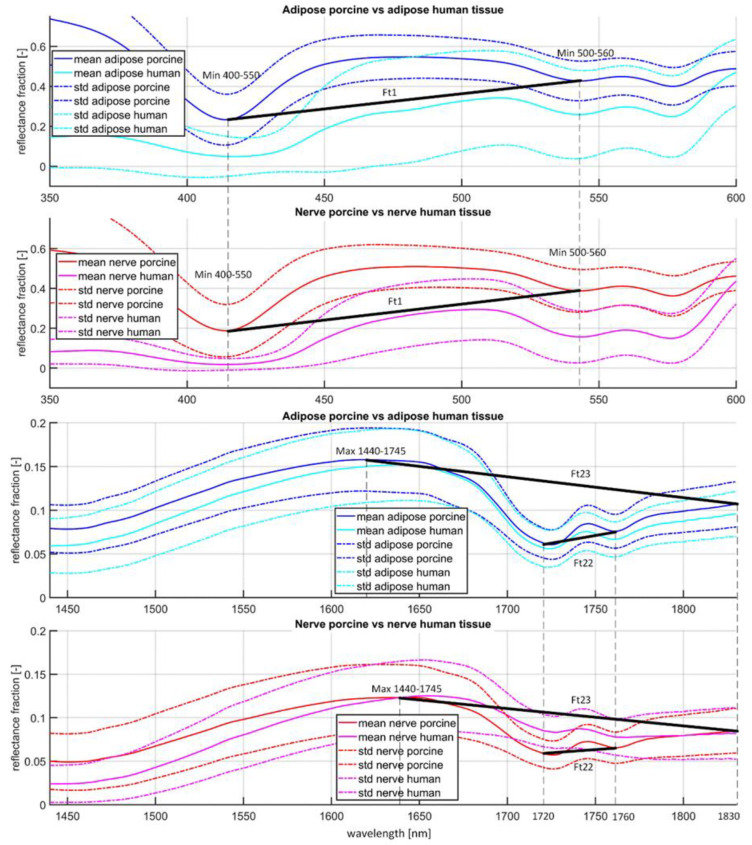
Two reproductions of Figure 3, but now zoomed in to wavelength range 350–650 (top 2 graphs) and zoomed in to wavelength range 1440–1830 (bottom 2 graphs). Mean calibrated spectra (solid lines) flanked by the standard deviation (striped lines). Porcine (blue) vs. human adipose tissue (cyan), and porcine (red) vs. human nerve tissue (magenta). The gradient features Ft1 (in top 2 graphs), and Ft22 and Ft23 (in bottom 2 graphs) were found to be most accurately predicting the human dataset.

The Kruskal–Wallis test assessed that 24 normalized features showed similar distributions (*p* > 0.05) between porcine and human features per adipose and per nerve tissue type: i.e., Ft1-Ft4, Ft6, Ft8-Ft11, Ft13, Ft16-18, Ft20, Ft22, Ft23, Ft27-Ft28, Ft30-Ft 36. The BLRMs trained with combinations of these 24 features resulted in six BLRMs with two features, seven BLRMS with three features, four BLRMS with four features and six BLRMS with five features. The best performing BLRMs for each set of non-normalized features (for two, three, four, or five features) showed accuracies of 80% and slightly higher as highest accuracies on the cross validation set (Figure 4). The test accuracy of these BRLMs, assessed on the human test set, was 60% for a three feature BLRM generated with Ft1, Ft22 and Ft23 (Figure 4). A 58% accuracy was found for all BLRMs with two features, the remaining BLRMs with three features and three BLRMs with four features. For reference, the gradient features Ft1, Ft22 and Ft23 are indicated in Figure 5. 

## 4. Discussion

The aim of this study was to assess whether ex vivo porcine tissue could generate substitute input data to train models for classification of in vivo human tissue types. To this end, we focused on assessing similarities between ex vivo porcine and in vivo human spectral data of nerve and adipose tissue and the performance of porcine input data to generate BRLMs to classify human nerve and adipose tissue. The results show that there are similarities between the ex vivo porcine and in vivo human data, but this is insufficient to perform classification with satisfactory accuracy (Figure 3 and Figure 4). 

In more detail, the NMCC values of ≥0.9 between porcine and human confirm high similarity in the shapes of the curves of the spectral data (Figure 3). However, the magnitudes of the spectral reflectance data show a clear difference between porcine and human, as expressed by the NRSME being > 0.1 (Figure 3). The difference in the wavelengths 350–600 nm can be explained by the blood related characteristics in this zone as represented by the so-called ‘W’-sign [23,27]. This shape indicates the presence of HbO_2_ and is a direct measure of the presence of oxygenated blood, which is minimal in the porcine samples, since they were recruited ex vivo, frozen, and thawed before measurement. For the wavelengths zone larger than 1350 nm, characteristic for the fat related spectral reflectance, the porcine and human more similar (Figure 3 bottom, Table 1) [24]. This is partly supported by the BRLM that performed best, which had two features (Ft22 and Ft23) located in this region (Figure 5). This interpretation is supported by the work of Nachabe and coworkers who indicated that in vivo swine adipose tissue qualified to estimate lipid concentrations for their computational spectroscopy model [12,28].

The distinction between adipose and nerve tissue of ex vivo porcine tissue is less present compared to the in vivo human dataset (compare Figure 2 and Figure 3). This similarity between ex vivo porcine adipose and nerve tissue is probably the main contributor in not providing added benefit when generating BLRM for tissue classification. This is confirmed by the accuracy of the cross validation of the BLRMS, which at best was 83.5%. The BLRMs of this current study built with the ex vivo porcine data give a lower accuracy on their own dataset compared to the 100% accuracy of the BLRMs built with the in vivo human tissue dataset [1,10,29]. Thus, the ability to discriminate between different types of tissues within the ex vivo porcine BLRM is inferior compared to the in vivo human tissue types. This partly explains the maximum accuracy of around 60% of the BLRMs of this study on the in vivo human validation dataset (Figure 4). A higher performance rate is not to be expected. Moreover, none of the features found in [1] (i.e., Ft5, Ft7, Ft12 and Ft26) were identified as predictive feature in the BLRMs generated with the porcine data. The ex vivo effect of the porcine tissue probably plays a role in this result. As suggested, this effect is most distinct for blood related features due lack of perfusion in ex vivo tissue (Figure 2) [23,27]. Nevertheless, the adipose and nerve tissue composition between in vivo human and ex vivo porcine tissue is comparable (Table 1) [15,16,17,18], also for analysis of the spectral reflectance [12,28,30,31]. A possible explanation for the increased similarity between porcine adipose and nerve tissue could be that, overall, the swine are fatter, with possibly increased lipid content in the nerves. Other explanations for the lower accuracy of the BLRMs are related to the limitations of this study. The most important one is the time-consuming nature of the sample preparation by dissection from the shoulder of the pigs, which forced us to freeze and subsequently thaw the ex vivo porcine tissue before measurements to preserve tissue integrity. This could have affected the ex vivo adipose tissue or affected adipose and nerve tissue at different levels. Other studies using ex vivo adipose tissue for spectral analysis as substitute for human tissue [30,31] did not freeze the tissue before use, but only cooled it. Although care was taken, it is possible that the porcine nerves were not completely exposed, with a remaining small layer of fatty tissue surrounding them. Furthermore, the limited resources on swine anatomy could have compromised the recruitment of adequate samples as all senses, including haptic senses, were required to identify the anatomic regions and from that recruit adequate samples. Some samples might have had a too small or too large thickness which could influence the reflectance pattern. As explained with Figure 1, utmost care was taken to prevent these limitations, and we recruited a large porcine dataset to minimize the influence of outliers. Finally, the use binary logistic regression models for classification were chosen to compare the results with our previous studies. However, as the spectral similarity is high for the majority of spectra acquired, more advanced machine learning techniques to deal with noisy labels, magnitude differences and overfitting or a combination of porcine and human data might give the desired classification performance. 

The results do indicate that in vivo porcine tissue might indeed be considered as a substitute for human adipose and nerve tissue as also used by others [12,28]. However, considering the claimed benefits of using ex vivo porcine tissue as being abundant, and availability, this does not hold for in vivo porcine tissue. Swine do not receive surgery on a regular basis and the availability of in vivo porcine data is scarcer than in vivo human data. An alternative future direction to recruit a sufficiently large dataset for human tissue classification would be to screen for technology that facilitates the spectral data recruitment in the operating room. One can think of contactless 2D spectral cameras that can recorded a large tissue area in one shot without the need to be in contact with the tissue.

## 5. Conclusions

Shape-wise spectral similarity is present between ex vivo porcine and in vivo human adipose and nerve tissue. The difference between ex vivo porcine adipose and nerve tissue was not sufficient to use as substitute training data for classification of in vivo human tissue. Further research is required before ex vivo porcine data can be used to enhance human tissue classification.

## Figures and Tables

**Table 1:** Comparison between human and porcine adipose and nervous tissue compositions of lipid, water, and fat using various sources in literature [15,16,17,18].

	Composition	Human [%]	Porcine [%]
Adipose	Lipid (adipose)	61–87	68
	Water	11–31	25
	Protein (collagen)	8	
Nerve	Lipid (adipose)	20	1–20
	Protein (collagen, fibroblasts)	80	81–95

## Data Availability

The data presented in this study are available on request from the corresponding author.
